# Kranz and single-cell forms of C_4_ plants in the subfamily Suaedoideae show kinetic C_4_ convergence for PEPC and Rubisco with divergent amino acid substitutions

**DOI:** 10.1093/jxb/erv431

**Published:** 2015-09-28

**Authors:** Josh J. Rosnow, Marc A. Evans, Maxim V. Kapralov, Asaph B. Cousins, Gerald E. Edwards, Eric H. Roalson

**Affiliations:** ^1^School of Biological Sciences, Washington State University, Pullman, WA 99164-4236, USA; ^2^Department of Mathematics, Washington State University, Pullman, WA 99164-3113, USA; ^3^School of Natural Sciences and Psychology, Liverpool John Moores University, Liverpool L3 3AF, UK

**Keywords:** Bienertia, C_4_ photosynthesis, PAML, phosphoenolpyruvate carboxylase, positive selection analysis, Rubisco, Suaedoideae.

## Abstract

The two carboxylation reactions performed by phosphoenolpyruvate carboxylase (PEPC) and ribulose-1,5-*bis*phosphate carboxylase/oxygenase (Rubisco) are vital in the fixation of inorganic carbon for C_4_ plants. The abundance of PEPC is substantially elevated in C_4_ leaves, while the location of Rubisco is restricted to one of two chloroplast types. These differences compared with C_3_ leaves have been shown to result in convergent enzyme optimization in some C_4_ species. Investigation into the kinetic properties of PEPC and Rubisco from Kranz C_4_, single cell C_4_, and C_3_ species in Chenopodiaceae s. s. subfamily Suaedoideae showed that these major carboxylases in C_4_ Suaedoideae species lack the same mutations found in other C_4_ systems which have been examined; but still have similar convergent kinetic properties. Positive selection analysis on the N-terminus of PEPC identified residues 364 and 368 to be under positive selection with a posterior probability >0.99 using Bayes empirical Bayes. Compared with previous analyses on other C_4_ species, PEPC from C_4_ Suaedoideae species have different convergent amino acids that result in a higher *K*_m_ for PEP and malate tolerance compared with C_3_ species. Kinetic analysis of Rubisco showed that C_4_ species have a higher catalytic efficiency of Rubisco (*k*_catc_ in mol CO_2_ mol^–1^ Rubisco active sites s^–1^), despite lacking convergent substitutions in the *rbcL* gene. The importance of kinetic changes to the two-carboxylation reactions in C_4_ leaves related to amino acid selection is discussed.

## Highlight

Enzyme kinetic measurements and positive selection analysis show that C_4_ species in Suaedoideae have PEPC and Rubisco kinetics similar to other C_4_ species despite different amino acid convergence.

## Introduction

When organisms develop the same solution to an abiotic or biotic stress resulting in a similar character state, it is referred to as convergent evolution or phenotypic convergence. One of the most documented convergent phenotypes in plants is the repeated development of C_4_ photosynthesis, an adaptation that uses four carbon acids to increase photosynthesis under conditions where carbon assimilation can be limited by high photorespiration (Sage *et al.*, 2012). The number of times that C_4_ independently developed (at least 66) makes it an extremely useful phenotype for analysing the genetics of adaptations (Christin *et al.*, 2010; Sage *et al.*, 2011).

The genetic mechanisms responsible for C_4_ photosynthesis remain largely unknown, but they are thought to involve co-ordinated changes to genes that affect leaf anatomy, cell ultrastructure, energetics, metabolite transport, and the location, content, and regulation of many metabolic enzymes (Hibberd and Covshoff, 2010). One approach to gain further insight into the underlying genetic regulation of C_4_ photosynthesis is to analyse how enzymes are optimized for C_4_ biochemistry.

In C_4_ plants there is spatial separation between the capture of atmospheric CO_2_ with synthesis of C_4_ acids, and the donation of CO_2_ to Rubisco by decarboxylation of C_4_ acids, which, in most species, occurs in mesophyll and bundle sheath (BS) cells, respectively. In the mesophyll cells, atmospheric CO_2_ is initially converted into bicarbonate (${\text{HCO}}_{3}^{-}$ ) by carbonic anhydrase and the chloroplasts generate phosphoenolpyruvate (PEP) from pyruvate by pyruvate, Pi dikinase. Then, in the cytosol, the ${\text{HCO}}_{3}^{-}$ and PEP are utilized as substrates for phosphoenolpyruvate carboxylase (PEPC, EC 4.1.1.31) for the synthesis of oxaloacetate (Chollet *et al.*, 1996). The oxaloacetate is subsequently reduced in the chloroplast to malate (MA) by NADP-malate dehydrogenase or transaminated to aspartate (Asp) by Asp aminotransferase. The MA and Asp are transported to BS cells where CO_2_ is donated to Rubisco via C_4_ acid decarboxylases. How C_4_ photosynthesis is regulated, by the level of enzymes and their kinetic properties, their state of activation, and their control by allosteric effectors, is important for understanding the mechanism and how they accomplish high rates of photosynthesis under CO_2_ limiting conditions.

The kinetic properties of PEPC and Rubisco from C_4_ plants are different from those in C_3_ plants, which are considered to have optimized their function in the C_4_ system (Ghannoum *et al.*, 2005; Gowik and Westhoff, 2011; Whitney *et al.*, 2011b). These differences have led to questions about how these changes occurred during the evolution of C_4_ from C_3_ by positive selection on certain amino acid residues. PEPC in C_4_ plants have high enzymatic activities, as much as 20–40-fold higher than C_3_ plants (per mg of chlorophyll), and *K*_m_ values for PEP are several fold higher than C_3_ plants (Ting and Osmond, 1973; Kanai and Edwards, 1999; Engelmann *et al.*, 2003; Lara *et al.*, 2006). The C_4_ PEPC can have cooperativity with PEP as substrate (reflected in higher Hill coefficients), be less sensitive to the inhibition of catalysis by Asp and MA, and react to the positive allosteric effectors glucose, 6-phosphate (G6P), glyceraldehyde-3P, and glycine (Gowik and Westhoff, 2011). G6P decreases the *K*_m_ for PEP (Engelmann *et al.*, 2003; Gowik *et al.*, 2006), and lowers the inhibition by MA (Gupta *et al.*, 1994; Chollet *et al.*, 1996; Engelmann *et al.*, 2003). Positive selection analysis to identify amino acid residues under selection, that may account for the observed kinetic properties of the C_4_ PEPC, have been made in family Asteraceae (in C_3_, intermediate, and C_4_ species in the genus *Flaveria*), Cyperaceae, and Poaceae (Christin *et al.*, 2007; Besnard *et al.*, 2009; Gowik and Westhoff, 2011). This includes the identification of amino acid substitution at residue 780 to a serine in the C_4_ species which has been considered a key substitution in PEPC for C_4_ kinetics.

Rubisco in C_4_ plants functions where the ratio of CO_2_ to O_2_ is elevated, resulting in a decrease of the oxygenase reaction with RuBP and photorespiration. The high CO_2_ concentration provides selective pressure for a faster turnover of the enzyme under saturating CO_2_, resulting in higher *k*_*catc*_ and *K*_m_(CO_2_) values (Yeoh *et al.*, 1981; Seemann *et al.*, 1984; Sage, 2002; Kubien et al., 2003, 2008; Ghannoum *et al.*, 2005). These kinetic changes to Rubisco in C_4_ plants allow for a reduced investment in the enzyme, as much as half as in C_3_ leaves, while achieving higher rates of photosynthesis under warm temperatures and current ambient levels of CO_2_ due to their CO_2_-concentrating mechanism (Long, 1999; von Caemmerer, 2013). Rubisco is a heterooctomer composed of multiple small and large subunits which are encoded by nuclear *RbcS* and chloroplast *rbcL* genes, respectively (Whitney *et al.*, 2011a). Analyses for *rbcL* amino acid residues under positive selection in C_4_ lineages have been made mainly in families Poaceae, Cyperaceae, and Amaranthaceae s.l. (Kapralov and Filatov, 2007; Christin *et al.*, 2008, 2009; Kapralov *et al.*, 2011, 2012).

Among eudicot families, Chenopodiaceae s.s. has the largest number of eudicot C_4_ species and the most diversity in forms of C_4_, yet there is no information comparing the kinetic properties of the carboxylases and positive selection of amino acid residues in C_4_ lineages. The focus of the current study was on subfamily Suaedoideae which has diverse forms of C_4_ along with C_3_ species (Edwards and Voznesenskaya, 2011; Kadereit *et al.*, 2012). There are four independent origins of C_4_ in the subfamily, including two distinct Kranz anatomies in *Suaeda* sections *Salsina* s.l. and *Schoberia*, and two independent origins of single-cell C_4_ anatomy, in *Suaeda aralocaspica* and in genus *Bienertia* (Kapralov *et al.*, 2006; Rosnow *et al.*, 2014). A recent positive selection analysis on C_4_ PEPC in Suaedoideae showed that there was divergence in where positive selection was occurring compared with previous studies in grasses and sedges (Rosnow *et al.*, 2014). In the current study, the kinetic properties of PEPC across C_3_ and C_4_ Suaedoideae species, including the affinity for PEP, the kinetic response to allosteric effectors (G6P and MA), and the degree of cooperativity with varying PEP as substrate, were investigated together with additional PEPC sequence information.

With respect to Rubisco, positive selection analysis for *rbcL* in Amaranthaceae s.l. showed evidence for selection of residues at positions 281 and 309 among C_4_ species, which has also been observed in C_4_ monocots (Kapralov *et al.*, 2012). Also a functional analysis with hybrids of Rubiscos utilizing *rbcL* genes from C_3_ versus C_4_ *Flaveria* species indicated that a substitution in the *rbcL* gene at position 309 from a methionine to an isoleucine results in a higher Rubisco *k*_catc_ (Whitney *et al.*, 2011b). However, the three Suaedoideae C_4_ species which were previously analysed (*Suaeda altissima, S. microphylla*, and *Bienertia cycloptera*) lacked substitutions at 281 and 309 (Kapralov *et al.*, 2012). This raises questions about Rubisco kinetics (*k*_catc_) and *rbcL* sequences in Suaedoideae C_4_ lineages.

In this study, kinetic properties and sequence information for PEPC and Rubisco from the subfamily Suaedoideae were analysed. The results show that the C_4_ species have divergent amino acid positive selection resulting in convergent C_4_-type kinetic properties for PEPC and Rubisco.

## Materials and methods

### Plant material

All plants used in this study were started from seed and grown in controlled environmental chambers (Econair GC-16; Bio Chambers). Seedlings were started under low light [100 photosynthetic photon flux density (PPFD; μmol quanta m^–2^ s^–1^)] and temperature conditions with a day/night temperature of 25/22 °C and a photoperiod of 14/10h. The plants were moved to high light and temperature conditions (1,000 PPFD, with a day/night temperature of 35/25 °C and a photoperiod of 14/10h) once well established. A few leaves, for each replication, were sampled from 2–6-month-old plants and used for kinetic analysis.

### Enzyme extraction

Chlorophyll content, the quantity of Rubisco binding sites for RuBP, and Rubisco and PEPC activities, were measured on flash-frozen leaves from plants exposed to at least 5h of light in the chambers, using a liquid-nitrogen-chilled mortar and pestle (the extraction included 250mg leaf tissue plus 1ml extraction buffer). For Rubisco assays the extraction buffer consisted of [100mM 4-(2-hydroxyethyl)-1-piperazinepropanesulphonic acid (EPPS, pH 8.0), 1mM EDTA, and 10mM dithiothreitol (DTT)]; preliminary tests showed no difference in activity with or without the protease inhibitor (Sigma Protease Inhibitor Cocktail,P9599). For PEPC assays, the extraction buffer consisted of [100mM 4-(2-hydroxyethyl)piperazine-1-ethanesulphonic acid (HEPES, pH 7.6), 1mM EDTA, 1mM sodium fluoride, and 10mM dithiothreitol (DTT)]. The PEPC extraction included 1mM sodium fluoride to prevent the possible action of phosphatases on the PEP carboxylase protein. The frozen leaf powder was homogenized in the extraction buffer and, prior to centrifugation, a portion of the extract was placed in 80% acetone for chlorophyll determination (Porra *et al.*, 1989). The extract was centrifuged at 10,000 *g* relative centrifugal force for 1min at room temperature; the supernatant was collected and placed on ice. In the case of extracts for analysis of PEPC, the supernatant was desalted in a cold Sephadex G-50 column pre-equilibrated with the extraction buffer (to remove low-molecular-weight metabolites including the allosteric effectors malate, aspartate, and G6P, as well as cations, which may affect the assay).

### PEPC kinetic assays

Assays were performed immediately following desalting, and there was no apparent loss in activity during the assay period. The activity was coupled to the MA dehydrogenase reduction of OAA and measured as a decrease in absorbance at 340nm resulting from the oxidation of NADH. The standard assay mixture contained 100mM HEPES–KOH (pH 7.6), 10mM MgCl_2_, 10mM NaHCO_3_, 0.2mM NADH, 12U NADH-MA dehydrogenase (MP Biomedicals), and 10 μl of enzyme extract in a total volume of 1ml. The reaction was started by the addition of PEP (with or without G6P as indicated). In order to determine the *K*_m_, *V*_max_, and Hill coefficient for PEP, the Hill equation was fitted to the experimental data by non-linear regression analysis with the software package KaleidaGraph 4.5 (Synergy Software):

$$V=\frac{V_{\text{max}}{\left[S\right]}^{\text{h}}}{K^{\text{n}}+{\left[S\right]}^{\text{h}}}$$

(where *V*, velocity; *V*_max_, maximum velocity, *K*, half maximum rate; *S*, Substrate PEP; and h, Hill coefficient).

For each species, two independent extractions were analysed and each kinetic measurement was repeated.

The *IC*_50_ for MA, the concentration causing 50% inhibition of PEPC activity, was determined using the coupled spectrometric assay as described above. For each species, the MA inhibition was measured at a PEP concentration which was twice the *K*_m_ (using the value of *K*_m_ determined in the presence or absence of G6P). Separate assays were performed with a range of MA concentrations from 0mM to 20mM. The MA *IC*_50_ values are from two independent biological replications, with two technical replications on each. The *IC*_50_ for MA of PEPC was calculated from the experimental data by fitting the same normalized three parameter dose–response curve using the following equation for all species using SAS Proc NLIN (SAS, 2011).

$$V=\frac{V_{\text{min}}+\left(100-V_{\text{min}}\right)}{1+10^{\left(\left(\text{S}-{\text{IC}}_{50}\right)\text{n}\right)}}$$

(where *V*, velocity; *V*_min_, minimum velocity; *IC*_50_, concentration of MA causing 50% inhibition; *S*, substrate PEP; *n*, slope at *IC*_50_). SAS Proc NLIN was coded so that the response equation was simultaneously computed for all 14 combinations of the seven species and two levels of G6P. This allowed for global tests of equality for *IC*_50_ between species and G6P levels.

### Rubisco *k*_catc_ analysis

From measurement of Rubisco catalytic sites and Rubisco activity, *k*_catc_ values were determined (mol CO_2_ mol^–1^ binding site s^–1^) (Lilley and Walker, 1974; Collatz *et al.*, 1979; Walker *et al.*, 2013). In the leaf extracts, Rubisco catalytic sites were quantified from the stoichiometric binding of radiolabelled ^14^C-carboxy-arabinitol-*bis*phosphate (^14^CABP). For ^14^CABP binding assays, 20 μl of enzyme extract was incubated in 150mM EPPS, 18mM MgCl_2_, 17.5mM ${\text{NaHCO}}_{3}^{},$ and 1mM ^14^CABP. A portion of the sample was then passed through a low-pressure chromatography column (737-4731; Bio-Rad, Hercules, CA, USA) packed with size exclusion beads (Sephadex G-50 Fine; GE Healthcare Biosciences, Pittsburgh, PA, USA). Samples were analysed in a liquid scintillation counter to quantify binding sites. Rubisco activity was determined spectrophotometrically. Rubisco activity was measured in 1ml of assay buffer (100mM EPPS pH 8.0, 20mM MgCl_2_, 1mM EDTA, 1mM ATP, 5mM creatine phosphate, 20mM NaHCO_3_, and 0.2mM NADH) containing coupling enzymes, (12.5U creatine phosphokinase, 250U carbonic anhydrase, 23U 3-phosphoglycerate kinase, 20U glyceraldehyde-3-phosphate dehydrogenase, 56U triose-phosphate isomerase, and 20U glycerol-3-phosphate dehydrogenase), and 10 μl of enzyme extract. Rubisco was activated for 10min at 25 °C, before the addition of coupling enzymes and initiation with 0.5mM RuBP. The activity of Rubisco was determined from the rate of conversion of NADH to NAD^+^, which was monitored by the change in absorbance at 340nm.

### DNA sequencing and analysis

PEPC and Rubisco large subunit (L-subunit) genes, *ppc-1* and *rbcL*, were sequenced for 17 *Suaeda* species and two *Bienertia* species. DNA was extracted from 250mg of plant material using the CTAB method following the protocol of Doyle and Doyle (1987). Primers were developed based on homology to previously published sequences (see Supplementary Table S1 at *JXB* online). Initial PCR conditions were 2min at 95 °C, followed by 35 cycles of: 30 s at 95 °C, 30 s at 52 °C annealing step, and a 3min extension at 72 °C. The PCR product was visualized and purified using a PCR clean-up kit according to the manufacturer’s protocol (Qiagen, USA). For ppc-1, purified PCR product was cloned into the pGEM T-easy vector using the manufacturer’s protocol (Promega, USA). Single colonies were grown overnight and plasmid DNA was purified using alkaline lysis with SDS (Sambrook and Russell, 2001). Plasmid inserts were PCR amplified using GOTaq (Promega, USA), Sp6 and T7 primers, and were visualized on a gel. Prior to sequencing, the PCR product was mixed with 2.5U of Antarctic Phosphatase and 4U of Exo-Sap Nuclease in Antarctic Phosphatase buffer (New England BioSciences, USA) to degrade primers and nucleotides, and subsequently diluted 1:10. Sequencing reactions were performed using the Big Dye terminator master mix v3.1 (Applied BioSciences, USA), using sequence specific internal primers along with Sp6 and T7 (see Supplementary Table S1 at *JXB* online). Sequencing was carried out at Washington State University genomics core. Sequence data was assembled using Sequencher software (USA). Nucleotide sequences were translated, aligned, and visualized using Se-Al and MacVector (USA). All sequences were deposited in GenBank (see Supplementary Table S2 at *JXB* online). Positive selection analysis on additional N-terminus PEPC residues was performed using the methodologies of Rosnow *et al.* (2014). The same phylogenetic tree from the previous study was used for selection analysis, but was pruned to exclude *S. heterophylla, Salsola genistoides,* and *Salsola divaricata* as these species were not sequenced in this study. Throughout this paper, the numbering of PEPC residues is based on the *Zea mays ppc-B2* sequence CAA33317 (Besnard *et al.*, 2003) for easy comparison with previous studies.

### δ^13^C determination

Measurements of carbon isotope fractionation values (δ^13^C) were made on all Suaedoideae species used for kinetic analysis to verify photosynthetic type (see Supplementary Table S3 at *JXB* online). Analyses were made at Washington State University on leaf samples taken from plants grown in growth chambers. A standard procedure relative to Pee Dee Belemnite (PDB) limestone as the carbon isotope standard (Bender, 1973). Plant samples were dried at 80 °C for 24h, then 1–2mg was placed in a tin capsule and combusted in a Eurovector elemental analyser. The resulting N_2_ and CO_2_ gases were separated by gas chromatography and admitted into the inlet of a Micromass Isoprime isotope ratio mass spectrometer (IRMS) for determination of ^13^C/^12^C ratios (*R*). δ^13^C values were determined where δ=1 000(*R*_sample_/*R*_standard_)−1.

### Statistical analysis

For PEPC kinetic parameters determined from varying response to PEP, the statistical design was completely randomized with a two-way treatment structure (nine species, with and without G6P). SAS Proc MIXED (SAS, 2011) was used to compute parameter estimates and test statistics. The assumption of equal variances was assessed and determined to have been violated for all three response variables (*K*_m_, Hill coefficient, and *V*_max_) with a *P*-value <0.0001. Because of this, the variances were modelled as part of the mixed model analysis that was used to assess the main effects of species and G6P, along with the interaction between species and G6P. Fisher’s LSD was used to assess pairwise comparison between means. In addition, contrasts were also used to assess whether differences existed between linear combinations of the cell means (average value for each parameter) as they related to the different photosynthetic modes and sequence types. In particular, the photosynthetic modes compared with contrasts were the Kranz C_4_, single-cell C_4_, and C_3_ representatives in Suaedoideae, and comparisons made with the monocot *Z. mays*. In addition, contrasts were also computed to compare species differences at PEPC residues 733 and 780. All comparisons were taken to be significant at the *P* <0.05 level.

For Rubisco *k*_catc_ values, one-way analysis of variance (ANOVA) was performed using Sigma-Plot version 11.0 software (Systat Software Inc.). Post-hoc analysis was used to test statistical significance. All comparisons were taken to be significant at the *P* <0.05 level.

## Results

### PEPC sequence analysis

To complement previous sequence and phylogenetic information on PEPC in Suaedoideae (Rosnow *et al.*, 2014), N-terminal PEPC sequence was obtained for 19 species (*S. heterophylla, Salsola genistoides,* and *Salsola divaricata* were not included) using homologous upstream primers that overlapped with known C-terminal *ppc-1* sequence. The region of coverage included part of exon 2 through exon 8, stopping where previous C-terminal sequence analysis had been performed (Rosnow *et al.*, 2014). The sequenced region resulted in an additional 370 N-terminal amino acids of the *ppc-1* coding sequence. Based on gene homology to previously sequenced *Alternanthera* species PEPCs (Gowik *et al.*, 2006), this is approximately 87 N-terminal amino acids short of complete *ppc-1* gene coverage.

Positive selection analysis, using phylogenetic relationships, models amino acid change identifying significant non-synonymous amino acid changes; for model descriptions see Rosnow *et al.* (2014) (Yang, 2007). There were no codons identified as being under positive selection with a posterior probability >0.95 by BEB in the M2A model or M8 model (see Supplementary Table S4 at *JXB* online) (*P* value=0.82 and 0.0071, respectively). There were seven codons (99, 171, 324, 333, 364, 365, 368) that were shown to be under positive selection with a posterior probability >0.95 by BEB, when only branches leading to C_4_ clades were labelled as foreground branches (*P* value <0.0001). Positions 364 and 368 were the only two residues identified to have a posterior probability >0.99 by BEB in Model A, when only branches leading to C_4_ clades were labelled as foreground branches (see Supplementary Table S4 at *JXB* online). Residues 364 and 368 are in the N-terminal region which is shown to be involved in the allosteric regulation of activators like G6P (Blasing *et al.*, 2002; Engelmann *et al.*, 2002; Takahashi-Terada *et al.*, 2005). Residue 364 had four alternative amino acids present in this dataset, Arg present in C_3_ species, and either Lys, Gln, or Pro in C_4_ species (in order of prevalence). Residue 368 has Asn present in C_3_ species and Ser in C_4_ species. Both residues had a substitution in all C_4_ species (see Supplementary Fig. S1 at *JXB* online). There were no codons shown to be under positive selection with a posterior probability >0.95 by BEB, when foreground branches leading to Kranz C_4_ clades or branches leading to single-cell C_4_ clades alone were labelled (see Supplementary Table S4 at *JXB* online) (*P* values=0.65 and 1, respectively). By labelling all C_4_ branches as foreground branches, 10 codons were identified as being under positive selection (157, 159, 171, 198, 314, 318, 324, 353, 364, 368) with a posterior probability >0.95 by BEB (see Supplementary Table S4 at *JXB* online) although the results are not significant (*P* value=0.22). Four of these residues (171, 324, 364, 368) were identified as being on branches leading to C_4_ clades.

### PEPC kinetics

**Fig. 1.**
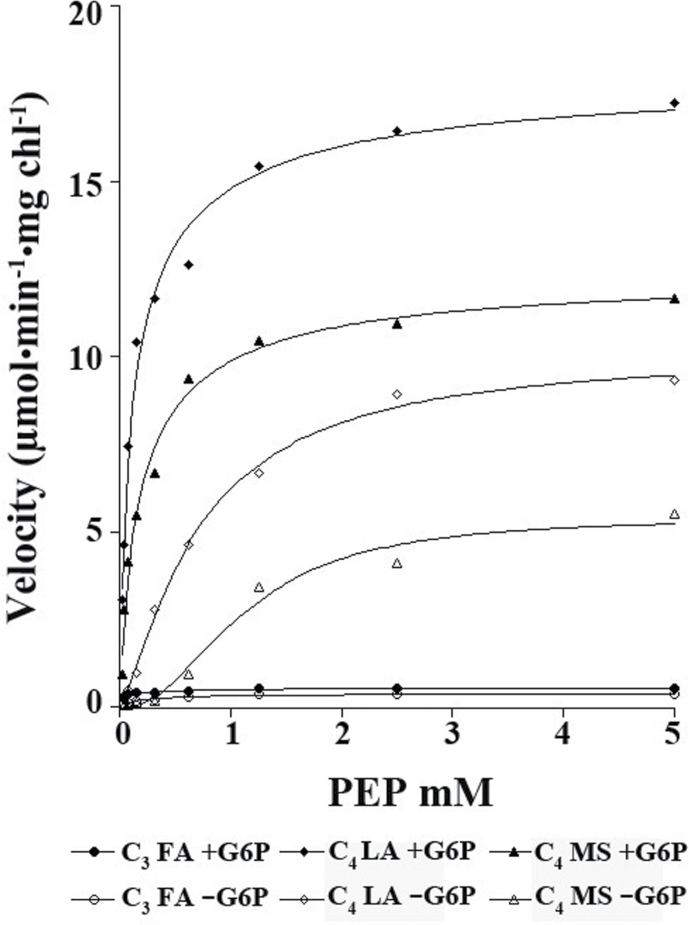
Representative PEPC kinetics based on photosynthetic mode (C_4_ or C_3_) and the amino acids present at residue 733 (M, L, or F) and residue 780 (A or S). PEPC rates were obtained at pH 7.6 with saturating Mg^2+^ and bicarbonate, while varying phosphoenolpyruvate (PEP) concentrations, either in the presence or absence of 5mM glucose-6-phosphate (G6P). The mean data points are presented for each PEPC type while species values are presented in Table 3. The solid line is the Hill equation fit to the data, see the Materials and methods for the equation details.

The *K*_m_ value for a given substrate in Michaelis–Menten kinetics is the concentration at which the rate of reaction is at half the maximal rate, which generally has an inverse relationship to affinity of enzyme for substrate. Since some forms of PEPC show cooperativity with PEP as substrate, the Hill equation was used to determine the *K*_m_, the Hill coefficient (*h*) for PEP, and *V*_max_. The analyses of PEPC kinetics in members of subfamily Suaedoideae representing different photosynthetic types, are shown in Tables 1–4 and Fig. 1. (see Supplementary Table S3 at *JXB* online for δ^13^C values of C_4_ and C_3_ species in the study)

**Table 1.** PEPC *K*_m_-PEP values (pH 7.6) in representative species in subfamily Suaedoideae Values were determined by curve-fitting the Hill equation to the data. Values represent the average of two biological and two technical replicates. The amino acid residues at positions 733 and 780, M (methionine), S (serine), L (leucine), A (alanine), F (phenylalanine), and V (valine) for the species are from Rosnow *et al.* (2014) and Besnard *et al.* (2003) for *Z. mays.*

Species	Photosynthetic	Amino acid at	Amino acid	*K*_m_ PEP (mM)		Hill coefficient (*h*)		Fold increase by G6P
	mode	residue 733	at residue 780	No G6P	5mM G6P	No G6P	+G6P	at 0.3mM PEP
*S. accuminata*	*Schoberia* Kranz C_4_	M	S	0.83±0.12	0.10±0.01	2.61±0.12	1.13±0.19	4.3
*S. eltonica*	*Schoberia* Kranz C_4_	M	S	1.04±0.14	0.14±0.02	2.71±0.24	0.90±0.08	2.7
*S. moquinii*	*Salsina* Kranz C_4_	L	A	0.46±0.12	0.14±0.01	1.45±0.06	0.96±0.07	3.2
*S. fruticosa*	*Salsina* Kranz C_4_	L	A	0.67±0.11	0.14±0.04	1.78±0.21	0.98±0.06	3.8
*S. aralocaspica*	Single-Cell C_4_	L	A	0.74±0.08	0.14±0.02	2.13±0.22	0.90±0.03	2.7
*S. linearis*	C_3_	F	A	0.21±0.01	0.03±0.01	1.19±0.15	1.29±0.25	2.3
*S. physophora*	C_3_	F	A	0.27±0.02	0.05±0.01	0.94±0.15	0.93±0.11	2.1
*S. linifolia*	C_3_	F	A	0.35±0.03	0.04±0.01	0.95±0.02	0.90±0.07	2.6
*Zea mays*	C_4_	V	S	1.23±0.07	0.15±0.08	1.18±0.01	1.17±0.07	5.0

**Table 2.** Contrasts of Suaedoideae PEPC kinetic parameters (*K*_m_ for PEP, Hill coefficient, and *V*_max_) under saturating Mg^2+^ and $HCO_{3}^{-}$ Contrasts were done based on amino acid residues 733 (M, L, or F) and 780 (S or A) or photosynthetic mode; see Table 1. In contrasts 1–3, amino acids are compared, MS (C_4_) occurs in *Schoberia* species, LA (C_4_) occurs in *Salsina* species and single-cell *S. aralocaspica*, and FA (C_3_) represents C_3_ species. In contrasts 4–9, the four photosynthetic modes are being compared, *Schoberia* type C_4_ (*S. eltonica* and *S. accuminata*), *Salsina* type C_4_ (*S. fruticosa* and *S. moquinii*), single-cell C_4_ (*S. aralocaspica*), and C_3_ species (*S. linearis*, *S. linifolia*, and *S. physophora*). *, Significant at the *P* <0.05 level of significance. (+) Indicates whether the first component of the contrast is larger than the second component and (–) indicates that the first component of the contrast is smaller than the second component. Maize data were excluded from analysis.

Contrast	*K*_m_		Hill coefficient		PEPC *V*_max_	
	No G6P	5mM G6P	No G6P	5mM G6P	No G6P	5mM G6P
1. MS (C_4_)×LA (C_4_)	*(+)	(–)	*(+)	(+)	(–)	(–)
2. MS (C_4_)×FA (C_3_)	*(+)	*(+)	*(+)	(+)	*(+)	*(+)
3. LA (C_4_) x FA (C_3_)	*(+)	*(+)	*(+)	(+)	*(+)	*(+)
4. *Schoberia* C_4_ (MS)×*Salsina* C_4_ (LA)	*(+)	(–)	*(+)	(+)	*(–)	*(–)
5. *Schoberia* C_4_ (MS)×SC C_4_ (LA)	(–)	(–)	(+)	(+)	*(–)	*(–)
6. *Salsina* C_4_ (LA)×SC C_4_ (LA)	(+)	(+)	(–)	(+)	(+)	(–)
7. *Schoberia* C_4_ (MS)×C_3_ (FA)	*(+)	*(+)	*(+)	(–)	*(+)	*(+)
8. *Salsina* C_4_ (LA)×C_3_ (FA)	*(+)	*(+)	*(+)	(–)	*(–)	*(+)
9. SC C_4_ (LA)×C_3_ (FA)	*(+)	*(+)	*(+)	(–)	*(+)	*(+)

**Table 3.** PEPC *V*_max_ values with saturating Mg^2+^ and $HCO_{3}^{-}$ for representative species in subfamily Suaedoideae at pH 7.6

Species	Photosynthetic mode	*V*_max_ (μmol min^–1^ mg^–1^ chl)		Fold increase in activity in presence of G6P
		No G6P	+ 5mM G6P	
S. accuminata	*Schoberia* Kranz C_4_	4.6	10.0	2.2
*S. eltonica*	*Schoberia* Kranz C_4_	6.5	12.9	2.0
*S. moquinii*	*Salsina* Kranz C_4_	9.9	15.6	1.6
*S. fruticosa*	*Salsina* Kranz C_4_	11.5	17.6	1.5
*S. aralocaspica*	Single-Cell C_4_	16.5	24.1	1.5
*S. linearis*	C_3_	0.1	0.4	3.5
*S. physophora*	C_3_	1.0	0.8	0.8
*S. linifolia*	C_3_	0.4	0.4	1.1
*Zea mays*	C_4_	15.1	21.1	1.4

**Table 4.** Estimates of malate *IC*_50_ values for half-maximum inhibition of PEPC activity at pH 7.6 (PEP concentration, 2× the *K*_m_) in representative photosynthetic types in subfamily Suaedoideae The amino acid residues potentially involved in malate tolerance are presented. For species comparisons, different letters indicate a significant difference within a category of G6P (+ or –) while comparison of G6P levels within a species is indicated by an asterisk (*) for significance at the *P* <0.05 level.

Species	Photosynthetic	Residue at	Residue at	Residue at	Residue at	*IC*_50_ (mM)	*IC*_50_ (mM)	Significant
	mode	780	868	879	890	No G6P	5mM G6P	effect of G6P
*S. accuminata*	*Schoberia* Kranz C_4_	S	R	D	R	0.6 c	1.4 c	*
*S. eltonica*	*Schoberia* Kranz C_4_	S	R	D	R	1.0 b	1.9 b	*
*S. moquinii*	*Salsina* Kranz C_4_	A	L	N	M	4.5 a	5.9 a	–
*S. fruticosa*	*Salsina* Kranz C_4_	A	L	N	M	4.5 a	5.2 a	–
*S. aralocaspica*	Single Cell-C_4_	A	Q	E	R	0.9 b	1.6 bc	*
*S. physophora*	C_3_	A	K	D	R	0.3 d	0.9 d	*
*S. linifolia*	C_3_	A	K	D	R	0.3 d	0.8 d	*

In Table 1, results are shown with PEPC for three forms of C_4_, Kranz-type *Schoberia* C_4_ (two species) Kranz-type *Salsina* C_4_ (two species), the single cell C_4_ *S. aralocaspica*, along with C_3_ type *Suaeda* (three species), and the C_4_ monocot *Z. mays*. Substitutions on amino acid residues at positions 733 and 780 are candidates for affecting the *K*_m_ for PEPC (Rosnow *et al.*, 2014). The four combinations among the *Suaeda* species for 733 and 780, respectively, are MS *Schoberia*, LA *Salsina*, LA single-cell C_4_, and FA C_3_ species. The results are shown from kinetic analyses for *K*_m_ PEP, the Hill coefficient, and the effect of the allosteric effector G6P. In Table 2, statistical analyses are shown for significant differences in *K*_m_ for PEP, the Hill coefficients, and the *V*_max_ of PEPC. Contrasts 1–3 in Table 2 show where there are differences in these parameters based on the three combinations of residues in *Suaeda* species at positions 733 and 780 (MS, LA, and FA). Contrasts 4–9 show where there are differences in these parameters based on the four photosynthetic types of *Suaeda.*

#### Absence of G6P

In kinetic analyses, in the absence of the allosteric effector G6P, the *K*_m_ values for PEP were higher in the three types of C_4_ species than in the C_3_ species (Table 1; Table 2, contrasts 7–9, *P* <0.05). Unlike the C_4_ species, the C_3_ species have a Phe (F) residue at position 733 (Table 1). Among the C_4_, the *K*_m_ PEP was significantly higher in Kranz *Schoberia* species (with a Met at 733 and a Ser at residue 780) compared with the Kranz *Salsina* species (with a Leu at 733 and an Ala at 780; Table 1; Table 2, contrast 4). In addition, the *K*_m_ PEP for MS *Schoberia* was significantly higher than the *K*_m_ PEP for the species having LA residues (*Salsina* C_4_ and single-cell C_4_; Table 2, contrast 1). There was no significant difference in the contrast between the single-cell C_4_ species (SC C_4_-LA) with either of the Kranz-type C_4_ species (Table 2, contrasts 5 and 6).

The cooperativity of PEPC for the binding of PEP was investigated by determining the Hill coefficient, from the curve fitting of the Hill equation to the data set. In the absence of G6P, the Hill coefficients were higher in C_4_ species (from 1.45–2.71) than in the C_3_ species (0.94–1.19) indicating cooperativity in binding of PEP in C_4_ species and no cooperativity in the C_3_ species (Table 1; Fig. 1; Table 2, contrasts 7–9). There was the same pattern of significant differences among species in the Hill coefficients as in the *K*_m_ for PEP. The C_4_ species had higher Hill coefficients than C_3_ species, the *Schoberia* C_4_ had higher Hill coefficients than the *Salsina* C_4_, while there was no significant difference in the coefficients between the single-cell C_4_ species *S. aralocaspica* and either Kranz C_4_ type (Table 2, contrasts 4–9).

#### Presence of G6P

In the presence of G6P there was a large decrease in the *K*_m_ for PEP in both C_4_ and C_3_ species (Table 1) which was significantly different in the absence of G6P (see Supplementary Table S5 at *JXB* online). This difference is highlighted by the fold increase in rate at 0.3mM PEP, where all species had at least a 2-fold increase in rate in the presence of G6P, with the highest increase in rate being found in C_4_ species (Table 1). The *K*_m_ values for PEP in the presence of G6P were higher in the C_4_ than in the C_3_ species (Table 1; Table 2, contrasts 2, 3, and 7–9). Among the C_4_ species there were no significant differences in *K*_m_ for PEP (Table 2, contrasts 1, and 4–6).

In the presence of G6P there was a large decrease in the Hill coefficient in the C_4_ species, whereas there was no significant difference in the C_3_ species with and without G6P (Table 1; see Supplementary Table S5 at *JXB* online). With G6P, in both C_3_ and C_4_ species the Hill coefficients were low (0.9–1.29) indicating little or no cooperativity in binding of PEP, and there was no significant difference in the coefficients between the photosynthetic groups (Table 2).

The change in *K*_m_ values for PEP in *Z. mays* with or without G6P was similar to that in the C_4_ *Suaeda* species. With the addition of G6P, the *K*_m_ PEP decreased from 1.23 to 0.15mM in *Z. mays*, with values in the presence of G6P similar to the C_4_ *Suaeda* species. Unlike the C_4_ *Suaeda* species, the Hill coefficients in *Z. mays* were low with and without G6P (~1.2) indicating no change in cooperativity with the allosteric effector (Table 1; see Supplementary Table S5 at *JXB* online).

#### *V*_max_

The maximum velocity (*V*_max_ on a chlorophyll basis) of the PEPC reaction was determined from the curve-fitting of the Hill equation; as expected, C_4_ species had much higher PEPC rates than C_3_ species (Table 3; Table 2, contrasts 7, 8, and 9). There was a significant increase in *V*_max_ for each C_4_ species in the presence of G6P, where the mean fold increase was 1.8 (Table 3; see Supplementary Table S5 at *JXB* online). In the C_3_ species, G6P had no significant effect on *V*_max_ in two of the C_3_ species; in C_3_ *S. linearis*, which had very low activity, there was some increase with G6P (Table 3; see Supplementary Table S5 at *JXB* online). Among the C_4_ contrasts, the *V*_max_ in the *Salsina* C_4_-LA is higher than *Schoberia* C_4_-MS, and SC C_4_-LA is higher than *Schoberia* C_4_-MS, while there is not a significant difference between *Salsina* C_4_-LA and SC C_4_-LA (Table 2, contrasts 4, 5, and 6).

Figure 1 shows the differences in activity in response to varying PEP for the different types of PEPC according to amino acid residues (LA type in *Salina* and SC-C_4_, MS type in *Schoberia*, and FA type for C_3_ species), with and without G6P. On a chlorophyll basis at high PEP, the C_4_ LA type has higher activity than the C_4_ MS type, while the C_3_ species have very low activity. Both the LA type and the MS type respond in a similar way. At 5mM PEP, the addition of G6P results in about a 2-fold increase in activity. In both types, at low levels of PEP, there is a large increase in activity with the addition of G6P as a consequence of lowering the *K*_m_ for PEP. This increase in activity by G6P at low PEP (e.g. at ~0.5mM PEP) is more dramatic in the MS type *Schoberia* (Fig. 1), which has a higher *K*_m_ for PEP and a higher Hill coefficient in the absence of G6P than the LA type (Table 1).

#### Malate inhibition

The concentration of a metabolic inhibitor that reduces the rate of an enzyme by 50% (*IC*_50_) is a useful determination in considering how *in vivo* metabolites might regulate enzyme activity. Table 4 shows the *IC*_50_ values for MA with species representing different photosynthetic types in Suaedoideae. Amino acid differences are shown for residues 868, 879, and 890, along with residue 780 which are candidates for affecting the *IC*_50_ for MA (Kai *et al.*, 2003; Paulus *et al.*, 2013b). There was a significant increase in the *IC*_50_ values in the presence of G6P in all species except in *Salsina*. The two *Salsina* C_4_ species had *IC*_50_ values that were significantly higher, with and without G6P, than any other species tested; the *Salsina* species also had different amino acid residues at position 868, 879, and 890. PEPC in the C_3_ species were the most sensitive to MA, both in the presence and absence of G6P. The two *Schoberia* C_4_ species that have Ser at residue 780 and Arg at residue 868, had *IC*_50_ values which were higher than the C_3_ species, but lower than *Salsina* C_4_ species (Table 4). The C_3_ species, which had the lowest *IC*_50_ values, were different from other species in having Lys at residue 868. The *IC*_50_ values for the single cell C_4_ *S. aralocaspica* were similar to the *Schoberia* type, and was different from other types in having a Gln residue at 868, and a Glu residue at 879. There was no significant difference in PEPC *IC*_50_ values based on the presence of a Ser versus an Ala residue at 780.

### Rubisco rbcL sequence information

A full-length *rbcL* sequence was generated for 20 Suaedoideae species, including at least two species from each Suaedoideae clade. There were 19 polymorphic Rubisco large subunit residues across the Suaedoideae species analysed, but none of the amino acid substitutions was invariantly fixed across C_4_ species (see Supplementary Table S6 at *JXB* online). Two C_3_ (*S. linifolia*, *S. vera*) and two C_4_ (*S. accuminata*, *S. aralocaspica*) species, representing four different sections, had identical amino acid sequences (see Supplementary Table S6 at *JXB* online). The PAML branch-site test for positive selection did not show significant evidence for selection along C_4_ branches (data not shown).

### Rubisco *k*_catc_

Measurement of Rubisco *k*_catc_ using the coupled enzyme assay showed that C_4_ species had significantly higher values than C_3_ species (Table 5). The average *k*_catc_ value for C_4_ was 2-fold higher than that of C_3_ species (3.6 versus 1.8mol CO_2_ mol^–1^ binding sites s^–1^). There was no significant difference in Rubisco *kcatc* between the single-cell C_4_ and Kranz species.

**Table 5.** Rubisco kcatc (mol CO_2_ mol^–1^ binding sites s^–1^) values for representative photosynthetic types in subfamily Suaedoideae One way analysis of variance a,b=statistically significant difference based on photosynthetic mode (C_3_ or C_4_) (*P* <0.05).

Species	Photosynthetic mode	Rubisco *k*_catc_	SD
*S. accuminata*	*Schoberia* Kranz C_4_	2.95	0.33
*S. eltonica*	*Schoberia* Kranz C_4_	3.34	1.18
*S. moquinii*	*Salsina* Kranz C_4_	3.76	0.30
*S. fruticosa*	*Salsina* Kranz C_4_	4.23	0.46
*S. altisima*	*Salsina* Kranz C_4_	3.19	0.17
	Mean Kranz C_4_	3.49 b	
*S. aralocaspica*	Single-Cell C_4_	3.77	0.39
*Bienertia cycloptera*	Single-Cell C_4_	3.90	0.27
*Bienertia sinuspersici*	Single-Cell C_4_	3.46	0.34
	Mean Single-Cell C_4_	3.71 b	
*Zea mays*	Kranz C_4_	3.58	0.00
*S. linearis*	C_3_	1.77	0.28
*S. physophora*	C_3_	1.52	0.09
*S. linifolia*	C_3_	2.08	0.17
*S. vera*	C_3_	1.82	0.49
	Mean C_3_	1.80 a	

## Discussion

### PEPC kinetic features in Suaedoideae: *V*_max_, affinity for PEP, regulation by G6P and MA

The maximum activities of PEPC (*V*_max_, μmol mg^–1^ chlorophyll) from leaves of the C_4_ *Suaeda* species were much higher than the C_3_ species, which is characteristic of C_4_ plants (Kanai and Edwards, 1999). In addition, compared with the C_3_ species, all of the C_4_ species analysed had a significantly higher *K*_m_ for PEP, both in the absence and presence of G6P (Tables 1, 2), which is the same general trend that has been reported throughout the literature (Svensson *et al.*, 1997; Gowik *et al.*, 2006; Lara *et al.*, 2006; Jacobs *et al.*, 2008). From studies in the genus *Flaveria* with *ppc-2*, the location of amino acids responsible for an increase in PEPC *K*_m_ was shown through reciprocal domain swapping to be in region 2 (amino acids 302–442) and region 5 (amino acids 651–966). In region 5, the single amino acid change to a Ser at residue 780 was suggested to be an important substitution resulting in the increase in *K*_m_ in C_4_ PEPC (Blasing *et al.*, 2000; Engelmann *et al.*, 2002). Subsequently, this substitution has been considered to be a key substitution for increasing the *K*_m_ PEP from analyses of various C_4_ species (Christin *et al.*, 2007; Besnard *et al.*, 2009; Gowik and Westhoff, 2011). However, the results of the current study, and from analysis of *Hydrilla verticillata* (a facultative aquatic C_4_ species) PEPC (Rao *et al.*, 2008), suggest that alternative substitutions can change the affinity for PEP. In Suaedoideae C_4_ species, a substitution at residue 733 in region 5 is a candidate for raising the *K*_m_, and the cooperativity in PEP binding (higher Hill coefficients).

Previous investigations on PEPC in C_4_ plants showed that the addition of phosphorylated sugars (e.g. G6P and triose-P) reduced the sigmoid nature of Michaelis–Menten kinetics plots, reducing the Hill coefficient to near one, demonstrating that allosteric activators can reduce the cooperative binding of PEP (Coombs and Baldry, 1975; Huber and Edwards, 1975; Nakamoto *et al.*, 1983; Bauwe and Chollet, 1986; Doncaster and Leegood, 1987; Tovar-Mendez *et al.*, 2000; Engelmann *et al.*, 2003; Gowik *et al.*, 2006). In addition, G6P has been shown to crystallize in the active site of the *Flaveria trinervia ppc-2* gene, demonstrating that it can also act as a competitive inhibitor (Schlieper *et al.*, 2014). In the present study, inclusion of the allosteric effector G6P in the assay of PEPC (pH 7.6) lowered the *K*_m_ for PEPC and increased enzyme activity in both the C_3_ and C_4_ species of *Suaeda.* However, in the absence of G6P, the C_4_ species showed cooperativity with PEP (the mean Hill coefficient for five species is 2.1) while the PEPC in C_3_ species showed no cooperativity (the mean Hill coefficient for three species is 1.0). This suggests certain substitutions in the C_4_ PEPC result in both an increase in *K*_m_ for PEP and an increase in the cooperativity of PEP binding.

Region 2 in the N-terminus was previously identified as the G6P regulatory site in C_4_ PEPC in *Z. mays* and it has also been suggested to influence the affinity of the enzyme for PEP (Kai *et al.*, 2003). In a study of representative photosynthetic types in *Flaveria*, residue 352 in region 2 of *ppc-2* (aka *ppcA*) was the only amino acid that showed differences between the C_4_ and C_4_-like *Flaveria* species which have a Lys residue, while the C_3_ and C_3_–C_4_ intermediate *Flaveria* have an Arg residue at this position. The C_4_ PEPC in *Z. mays* also has a Lys at residue 352 (Engelmann *et al.*, 2003). By contrast, current analysis of the N-terminus in *Suaeda* species showed position 352 is either a Thr or Ser residue (see Supplementary Fig. S1 at *JXB* online), and this residue is also an invariant Thr across *Alternanthera* PEPCs (Gowik *et al.*, 2006). In the *Suaeda* species, positive selection was found in region 2 at residues 364 (for Gln) and 368 (for Ser; see Supplementary Table S5 and Supplementary Fig. S1 at *JXB* online). The *Alternanthera ppc-1* gene has positive selection for Ser at residue 368, while residue 364 is invariant. Interestingly, the *ppc-1* gene in *Z. mays* and the *ppc-2* gene of *F. trineriva* (C_4_) has Asn at residue 368 (the same residue observed in all Suaedoideae C_3_ PEPC), while *F. pringeli* (C_3_) has Ser (the same amino acid observed in all Suaedoideae C_4_ PEPC). These results suggest that paralogous genes (*ppc-1* versus *ppc-2*) have undergone different selection processes. In C_4_ Suaedoideae and C_4_ *Alternanthera ppc-1*, substitution at residue 368 is a candidate for affecting the cooperativity with PEP as substrate, and regulation by binding G6P as an allosteric effector.

From species surveyed in Suaedoideae, the *IC*_50_ values for MA indicate that it is an effective inhibitor of PEPC at mM levels (Table 4). PEPC from C_3_ *Suaeda* species was more sensitive to inhibition by MA (assayed either with or without G6P) which is consistent with other studies where C_4_ PEPCs are generally reported to be more tolerant to MA compared with C_3_ orthologous genes or paralogous genes (Svensson *et al.*, 1997; Dong *et al.*, 1998; Blasing *et al.*, 2002; Paulus *et al.*, 2013a). Among the C_4_ *Suaeda*, the two *Salsina* Kranz species, had significantly higher *IC*_50_ values indicating higher tolerance to MA (when assayed in the presence or absence of G6P), compared with the *Schoberia* Kranz species and the single-cell C_4_ species *S. aralocaspica.* Also, with the addition of G6P, the *IC*_50_ for MA increased in the C_4_ *Schoberia* and C_3_ species, but not in the C_4_ *Salsina*. Studies on *Z. mays* show C_4_ PEPC has an allosteric site that binds MA and Asp, which is so close to the catalytic site that these metabolites act competitively with the substrate PEP, resulting in a less active enzyme (Izui *et al.*, 2004). In Suaedoideae, an amino acid substitution at residue 868 (Leu) is observed in all C_4_ species studied in the subfamily except *Bienertia* (see Supplementary Fig. S1 at *JXB* online) which may explain the difference in *IC*_50_ values between C_3_ and C_4_ species. The high *IC*_50_ values for MA in the *Salsina* species may be linked to their PEPC having, in addition to substitution at 868, substitutions at 879 (Asp), and 890 (Met) which is different from the other *Suaeda* species based on previous C-terminal PEPC sequence information (Rosnow *et al.*, 2014). Amino acid substitution at 868 is also observed in *Z. mays* and other Amaranthaceae C_4_ *ppc-1* genes, but not *Flaveria* C_4_ *ppc*-2. In other studies on MA inhibition of PEPC, a substitution at residue 884 from an Arg to a Gly in *Flaveria* was recently shown to increase tolerance to MA (Paulus *et al.*, 2013b). This substitution is also observed in some, but not all, C_4_ grass species (Paulus *et al.*, 2013a). However, this substitution is not observed in any C_4_ Suaedoideae species (Rosnow *et al.*, 2014). Using heterologously expressed chimeric *ppc-2* enzymes from *Flaveria*, the replacement of Ala 780 by Ser caused a slight increase in MA tolerance (observed in the presence, but not in the absence of G6P), which was not considered as the main determinant for higher MA tolerance in C_4_ PEPC (Jacobs *et al.*, 2008). In the present study, the highest tolerance to MA was in the *Salsina* C_4_ species, which have an Ala 780 and which also indicates other residues are the main determinants of tolerance.

The lack of strong convergence for a substitution near the MA/Asp allosteric pocket, suggests that there is less selective pressure on increasing MA tolerance than on increasing the *K*_m_ for PEP (decreased affinity), and G6P activation. Tolerance to MA may increase with alternative substitutions at different residues, without convergent amino acids, together with G6P activation and phosphorylation of PEPC in the light reducing sensitivity to MA. In the light, C_4_ PEPC is regulated by phosphorylation at a conserved N-terminus Ser residue, which leads to activation of the enzyme by reducing its sensitivity towards the allosteric inhibitors MA and Asp (Jiao and Chollet, 1991; Vidal and Chollet, 1997; Nimmo, 2003).

The current results raise questions about the molecular route for a C_4_ PEPC to acquire modified kinetic properties; i.e. modification to the allosteric activator site (residue 364/368) before or after increasing PEP *K*_m_ near the reaction site (733/780), and the impact of the order of mutations on selective pressure. Further analyses are needed to address the influence of amino acid substitutions on PEPC tolerance to MA in C_4_ Suaedoideae and other C_4_ species, versus the impact of *in vivo* phosphorylation of PEPC in the light (in this study extractions were made in the light).

Overall, the results indicate that phylogenetically distant C_4_ origins can optimize PEPC with divergent amino acid substitutions. The kinetic properties of C_4_ PEPC are considered to be optimized for function in C_4_ photosynthesis without interference with other metabolic processes. During the day, the positive allosteric effectors triose-P, G6P, and glycine are produced during photosynthesis in C_4_ plants (Leegood and von Caemmerer, 1988, 1989; DeVeau and Burris, 1989; Zelitch *et al.*, 2009). These positive allosteric effectors increase the affinity of PEPC for PEP and its effective use in the C_4_ cycle while the *IC*_50_ values for the C_4_ acids MA and Asp increases, which minimizes inhibition by products of C_4_ photosynthesis. Activity of PEPC at night can be controlled by the enzyme having a high *K*_m_ for PEP, due to relatively low levels of positive allosteric effectors (G6P, triose-P, and glycine) and by the non-phosphorylated form of the enzyme at night having a low *IC*_50_ for C_4_ acids (Doncaster and Leegood, 1987).

### Convergent evolution of Rubisco kinetics in C_4_ Suaedoideae achieved via non-parallel amino acid substitutions

In the current study of Suaedoideae, the determination of Rubisco *k*_catc_ showed that the enzyme in C_4_ species representing four lineages has, on average, approximately 2-fold higher catalytic rates than the C_3_ species. The mean *k*_catc_ value for these C_4_ species (all NAD-ME type), are similar to those of NAD-ME type C_4_ grasses (Ghannoum *et al.*, 2005). Although the *k*_catc_ values in Suaedoideae are higher for the C_4_ than the C_3_ species, sequence analysis of *rbcL* did not show any evidence for positive selection across lineages which could account for this adaptation.

The C_4_ species *S. aralocaspica* (section *Borszczowia*) and *S. accuminata* (section *Schoberia*) and the C_3_ species *S. linifolia* (section *Schanginia*) and *S. vera* (section *Suaeda*) have Rubisco large-subunit sequences that are identical (see Supplementary Table S6 at *JXB* online). This suggests that, in some C_4_ species, Rubisco with higher specific activity evolved via amino acid changes in the Rubisco small subunits. Positive selection on the small subunit encoding *RbcS* gene has previously been demonstrated for C_4_ *Flaveria*, which was strongly correlated with higher *k*_catc_ values and weakly correlated with higher *K*_m_(CO_2_) values (Kapralov *et al.*, 2011). Although Rubisco catalytic sites are located within the large subunits, significant changes in kinetics were shown when small subunits from C_3_ rice were replaced with those from C_4_ *Sorghum*, suggesting a differential role of S-subunits in Rubisco kinetics (Ishikawa *et al.*, 2011). Further work is necessary to determine if there are amino acids encoded by certain *RbcS* genes that are under positive selection, and candidates for determinant of the higher *k*_catc_ values in some Suaedoideae C_4_ lineages.

In C_4_ species representing sections *Salsina* and *Bienertia*, analysis of the Rubisco large-subunit residue polymorphism indicates that there are differences in amino acid residues compared with C_3_ species which may be associated with increased Rubisco *k*_catc_ values. The two single-cell C_4_ species from the genus *Bienertia* have three amino acid replacements putatively associated with increased Rubisco *k*_catc_. These are Ile 225, reported among submerged aquatic macrophytes (Iida *et al.*, 2009) which may be linked to kinetic properties of Rubisco associated with CO_2_-concentrating mechanisms (Yeoh *et al.*, 1981); Ile 270 which has been shown to be under positive selection along C_4_ lineages in Poaceae and Cyperaceae (Christin *et al.*, 2008); and Ser 439, found to be under positive selection among terrestrial species representing different phylogenetic lineages (Galmes *et al.*, 2014). Three Kranz C_4_ species from the section *Salsina* (see Supplementary Table S6 at *JXB* online) have Val 145 which is under positive selection along C_4_ lineages in Poaceae and Cyperaceae (Christin *et al.*, 2008). In addition, *S. altissima* from this section has Ile 270, suggesting parallel acquisition of this mutation with *Bienertia* species; while *S. moquinii* has Ser 281 (see Supplementary Table S6 at *JXB* online), which is reported to be under positive selection along C_4_ lineages in Poaceae and Cyperaceae (Christin *et al.*, 2008) and C_4_ lineages in Amaranthaceae (Kapralov *et al.*, 2012).

## Conclusions

The likelihood of a gene being repeatedly and independently recruited and changed to develop a convergent phenotype is most likely linked to the tissue in which it is expressed and its optimization for catalytic regulation. At the molecular level, when gene families are either recurrently recruited or when there are identical amino acid replacements in distant lineages, it suggests that there is limited genetic material suitable for new functions or that there is a restricted number of substitutions which can confer specific enzymatic properties (Christin *et al.*, 2010). For PEPC, the differences in substrate affinity and the reaction towards allosteric effectors, suggest that C_4_ PEPC’s harbour specific C_4_ determinants that were acquired during the evolution of C_4_ photosynthesis (Gowik and Westhoff, 2011). The results presented here suggest that the development of C_4_ photosynthesis can occur with divergent amino acid substitutions that alter enzyme kinetics to converge on the same function. To our knowledge this is the first report to demonstrate that PEPC from C_4_ terrestrial plants without Ser at position 780 have C_4_-like PEPC kinetics (and to identify candidates for positive selection at positions 364, 368, and 733). Similarly, this is the first case which shows that there are C_4_ species which have C_4_-type Rubisco *kcatc* values while lacking amino acid substitutions in the large subunit of Rubisco. This demonstrates that there are multiple molecular routes to the same C_4_ carboxylase phenotype.

## Supplementary data

Supplementary data can be found at *JXB* online.

Supplementary Table S1. Name and sequence of primers used.

Supplementary Table S2. Species origin, voucher, and sequence accession numbers.

Supplementary Table S3. Carbon isotope fraction values for leaf biomass.

Supplementary Table S4. *ppc-1*positive selection results.

Supplementary Table S5. Statistical analysis for PEPC *K*_m_ for PEP, Hill coefficient, and *V*_max_.

Supplementary Table S6. Rubisco large subunit residue polymorphisms.

Supplementary Fig. S1. Phylogeny of Suaedoideae taxa used for *ppc-1* positive selection analysis showing key amino acid changes.

Supplementary Data
