# Supplementary material for: Kranz and single-cell forms of C4 plants in the subfamily Suaedoideae show kinetic C4 convergence for PEPC and Rubisco with divergent amino acid substitutions
Source: J Exp Bot. 2015 Sep 28;66(22):7347–58. doi: 10.1093/jxb/erv431 (PMC4765798; doi:10.1093/jxb/erv431)
Supplement: Supplementary Data [file supp_66_22_7347__index.html]

Kranz and single-cell forms of C4 plants in the subfamily Suaedoideae show kinetic C4 convergence for PEPC and Rubisco with divergent amino acid substitutions — Kranz and single-cell forms of C4 plants in the subfamily Suaedoideae show kinetic C4 convergence for PEPC and Rubisco with divergent amino acid substitutions — Supplementary Data 

# Kranz and single-cell forms of C4 plants in the subfamily Suaedoideae show kinetic C4 convergence for PEPC and Rubisco with divergent amino acid substitutions

## Supplementary Data

Data files

- Supplementary Data - Supplementary Data
