# Supplementary material for: Kranz and single-cell forms of C4 plants in the subfamily Suaedoideae show kinetic C4 convergence for PEPC and Rubisco with divergent amino acid substitutions
Source: J Exp Bot. 2015 Sep 28;66(22):7347–58. doi: 10.1093/jxb/erv431 (PMC4765798; doi:10.1093/jxb/erv431)

Kranz and single-cell forms of C_4 plants in subfamily Suaedoideae show kinetic C_4 convergence for PEPC and Rubisco with divergent amino acid substitutions

Josh J. Rosnow · Marc A. Evans · Maxim V. Kapralov · Asaph B. Cousins · Gerald E. Edwards · Eric H. Rolason[1]

Supplementary Material 1 of 1.

Table S1. Name, sequence of primer, and which species the primer was used for Suaedoideae *ppc-1* and *rbcL*. S. = *Suaeda*, B. = *Bienertia*.

Primer Name	Primer Sequence 5' --> 3'	Species ppc-1 gene sequenced using this primer
SP6	ATTTAGGTGACACTATAG	All
T7	TAATACGACTCACTATAGGG	All
ppcex2_1365FW	ATTACCCCTGATGATAAGCAAGAGCT	*S. accuminata, S. aegyptiaca, S. altissima, S. aralocaspica, S. calceoliformis, S. eltonica, S. fruticosa, S. linearis, S. linifolia, S. moquinni, S. taxifolia, S. vera, S. vermiculata*
ppcex1_348FW	GCTCATCCAACTCAATCTGTTC	*S. accuminata, S. aegyptiaca, S. altissima, S. calceoliformis, B. cycloptera, S. eltonica, S. fruticosa, S. linearis, S. moquinni, S. nigra, S. occientalis, S. physophora, S. taxifolia, S. vermiculata*
*bs*PEPC-aE4FW930	AGCTGCATTTCGTACTGATG	*S. accuminata, S. aegyptiaca, S. aralocaspica, B. cycloptera, S. linearis, S. linifolia, S. maritima, S. nigra, S. occientalis, B. sinuspersici, S. vera*
ppcex6_665RV	GCAAGAACAACCCGATAAGGCTCA	*S. accuminata, S. aegyptiaca, S. calceoliformis, B. cycloptera, S. eltonica, S. fruticosa, S. linearis, S. linifolia, S. maritima, S. occientalis, S. physophora, S. vera,*
*bs*PEPC-aE7RV	ATAGCATAGCTCAAGAGGCTCC	*S. accuminata, S. aegyptiaca, S. altissima, S. aralocaspica, S. calceoliformis, S. eltonica, S. linearis, S. moquinii, S. nigra, S. occientalis, S. physophora, B. sinuspersici, S. taxifolia, S. vera, S. vermiculata*
LinePEPCA_527RV	CCAAATCTCCTTTCTTCAGTTTTTT	*S. aegyptiaca, S. altissima, S. occientalis,*
ppcex2_842RV	ATCCCATACCTCTCTTTGGAGAGC	*S. aegyptiaca, S. fruticosa, S. linifolia, S. physophora,*
ppcex2_1173FW	CAGGATGAGATGAGGGCAGGA	*S. aegyptiaca, B. cycloptera, S. maritima,*
ppcex6_665FW	TGAGCCTTATCGGGTTGTTCTTGC	*S. aegyptiaca, S. altissima, S. aralocaspica, S. calceoliformis, B. cycloptera, S. eltonica, S. fruticosa, S. linearis, S. linifolia, S. moquinii, S. occientalis, S. physophora, S. taxifolia, S. vera, S. vermiculata*
ppcex6_278RV	TTTCATACCTCAAACATGAGGTCCTG	*S. altissima, S. aralocaspica, S. calceoliformis, S. fruticosa, S. nigra, S. taxifolia,*
ppcex2_1365RV	AGCTCTTGCTTATCATCAGGGGTAAT	*S. aralocaspica, S. eltonica, S. fruticosa, S. linearis, S. nigra, S. taxifolia, S. vera,*
ppcex2_1082FW	CTGATGAAATCAGGAGGACTCAGC	*S. aralocaspica, S. linearis, S. linifolia, S. maritima, S. occientalis,*
ppcex1_689RV	TGAGCGGTAAGAACCAAGTCAACAGT	*S. calceoliformis, S. linearis, S. maritima,*
*bs*PEPC-aE7FW	GGAGCCTCTTGAGCTATGCTAT	*S. calceoliformis,*
ppcex2_842FW	GCTCTCCAAAGAGAGGTATGGGAT	*B. cycloptera, S. linifolia, S. nigra, S. physophora,*
ppcex6_524RV	ATAAAACTAAATGGGAATATTTGC	*S. fruticosa, S. moquinii, S. nigra, S. taxifolia, S. vermiculata*
ppcex6_278FW	CAGGACCTCATGTTTGAGGTATGAAA	*S. fruticosa, S. linifolia, S. maritima, S. nigra,*
ppcex6_524FW	GCAAATATTCCCATTTAGTTTTAT	*S. fruticosa, S. taxifolia,*
ppcex1_186FW	GATTTGAAGAAATCCCCTGAAGAG	*S. maritima,*
ppcex2_1173RV	TCCTGCCCTCATCTCATCCTG	*S. maritima, S. taxifolia,*
ppcex1_344RV	ATCTACGAACAGATTGAGTTGGATGAGC	*S. moquinni, S. nigra, S. physophora, S. taxifolia, S. vera, S. vermiculata*
*bs*PEPC-aRV1228	TTGGATTTCCTGCAGTCCATG	*B. sinuspersici,*
*bs*PEPC-aI6RV	CATTTTTCTTCACCAGTGAA	*B. sinuspersici,*
*bs*PEPC-aI5FW	TTCTTGTGGTATTGATTGAA	*B. sinuspersici,*
		Species *rbcL* gene sequenced using this primer
RbcL-49FW	CATTAAGGCCTAATTTATGTCGAGTAGACC	All
RbcL+1186FW	GATGATTCTGTACTACAGTTTGGTGGAGGAAC	All
RbcLStopRV	CTAGACTGTATCCATGGCTGGG	All
RbcL-Stop+3RV	CAACCGAACATTAATTACTTAGC	All
RbcL-1460-R	GTACCAGTAGAAGATTCGGCAG	All
RbcL-507-F	TATTGGGATGCACTATTAAAC	All
RbcL-1024-R	ATCAACAAAGCCTAAAGTAAT	All
RbcL-955-F	CGTCTGTCTGGTGGAGA	All

Table S2. List of species of Suaedoideae in this study, origin, voucher, and *rbcL*, *ppc-1* sequence accession numbers.

Species	Origin	Voucher	*rbcL*	*ppc-1*
Bienertia cycloptera	Kavir Protected Area near Mobarakiyeh, Iran	WS386420	KR057185	KT361188
Bienertia sinuspersici	Kuwait, collected by Abdulrahman Alsirhan	WS386421	KR057186	-
Suaeda acuminata	Armenia, collected by Maria Lomonosova	WS386425	KR057187	KT361187
Suaeda aegyptiaca	Kew, 011776	WS369795	KR057188	KT361186
Suaeda altissima	Armenia, collected by Maria Lomonosova	WS386426	KR057189	KT361185
Suaeda aralocaspica	Kazakhstan	WS369789	KR057190	KT361183
Suaeda calceoliformis	USA, Nevada, from H. Freitag	WS386422	KR057191	KT361184
Suaeda eltonica	Western Kazakhstan	WS369797	KR057192	KT361173
Suaeda fruticosa	Pakistan	WS386427	KR057193	KT361178
Suaeda heterophylla	United States	WS369803	KR057194	-
Suaeda linearis	USA, New Jersey, from H. Freitag	WS386424	KR057195	KT361182
Suaeda linifolia	United States	WS369788	KR057196	KT361181
Suaeda maritima	Kew, 59651	WS369798	KR057197	KT361180
Suaeda moquinii	Kew 0204473	WS386429	KR057198	KT361177
Suaeda nigra	GRIN, W6 27337	WS386428	KR057199	KT361176
Suaeda occientalis	USA, Nevada, from H. Freitag	WS386423	KR057200	KT361179
Suaeda physophora	Lake Elton, Soljanka river. Russia	WS386430	KR057201	KT361175
Suaeda taxifolia	United States, California	WS369802	KR057202	KT361174
Suaeda vera	Kew, 0083962	WS386431	KR057203	KT361172
Suaeda vermiculata	Kew, 0118549	WS369796	KR057204	KT361171

Table S3. Carbon isotope fraction values for leaf biomass (δ^{13}) from species used for enzyme kinetic analysis. The range of $\delta^{13}C$ values for the C_3 species was -32.9 ‰ to -28.9 ‰ (average -30.95 ‰), indicating high discrimination against ^{13}C isotope characteristic of C_3 plants. The range of $\delta^{13}C$ values for C_4 species was -15.9 ‰ to -19.0 ‰ (average -16.56 ‰), showing less discrimination against the ^{13}C isotope in C_4 plants.

Species	Photosynthetic mode	δ^{13}	S.D.
S. accuminata	Schoberia Kranz C_4	-15.9	0.5
S. eltonica	Schoberia Kranz C_4	-16.2	0.3
S altisima	Salsina Kranz C_4	-15.9	0.5
S. moquinii	Salsina Kranz C_4	-16.2	0.5
S. fruticosa	Salsina Kranz C_4	-19.0	0.6
S. aralocaspica	Single-Cell C_4	-16.5	0.9
Bienertia sinuspersici	Single-Cell C_4	-16.2	3.9
Zea mays	Kranz C_4	-13.8	0.6
S. linearis	C_3	-28.9	1.6
S. physophora	C_3	-32.9	0.5
S. linifolia	C_3	-30.4	0.6
S. vera	C_3	-31.6	0.4

Table S4. Positive selection analysis on *ppc-1* N-terminal residues 87-457 on species in subfamily Suaedoideae used in this study. [a]M1a (nearly neutral), M2a (positive selection), M8a (beta & ω= 1) and M8 (beta & ω) are PAML site models; A1 and A are PAML branch site models. [b] κ is transition/transversion rate ratio; ω_0 is dN/dS ratio; ω_s is dN/dS ratio in a class under putative positive selection; p_0 and p_s are proportion of codons with $\omega<1$ and $\omega>1$, respectively; p and q are parameters of beta distribution in the range (0, 1). [c] Sites listed are those at which positive selection is detected at the significance level of >95% or >99% in bold italics. [d] LRT is likelihood ratio test, 2l is twice the difference of model log-likelihoods. For additional model details see Rosnow et al. 2014 and Yang 2007.

Model with positive selection [a]				Null model [a]				LRT [d]
Model	log-likelihood	Parameters [b]	Positively selected sites [c]	Model	log-likelihood	Parameters [b]	2L	P-value
Analysis for positively selected sites common for C_3 and C_4 clades								
M2a	-3928.8	κ=2.36, p_0=0.9, ω_0=0.13, p_s=0.1, ω_S=1.16	none	M1a	-3929.0	κ=2.34, p_0=0.88, ω_0=0.12	0.4	0.8187
M8	-3928.8	κ=2.36, p_0=0.9, p=0.15, q=99, ω_S=1.18	none	M8a	-3932.4	κ=2.35, p=0.36, q=1.21	7.3	0.0071
Analysis for positive selection along branches leading to C_4 clades								
A	-3913.8	κ=2.36, p_0=0.82, ω_0=0.11, p_s=0.01, ω_S=3.9	99, 171, 324, 333, ***364,*** 365, ***368***	A1	-3921.1	κ=2.3, p_0=0.7, ω_0=0.1	14.7	0.0001
Analysis for positive selection along branches leading to Kranz C_4 clades								
A	-3918.8	κ=2.38, p_0=0.64, ω_0=0.06, p_s=0.03, ω_S=1.0	none	A1	-3918.7	κ=2.34, p_0=0.40, ω_0=0.04	0.2	0.6547
Analysis for positive selection along branches leading to Single Cell C_4 clades								
A	-3924.1	κ=2.36, p_0=0.74, ω_0=0.1, p_s=0.02, ω_S=1.0	none	A1	-3924.1	κ=2.36, p_0=0.71, ω_0=0.08	0	1
Analysis for positive selection along all C_4 branches								
A	-3909.7	κ= 2.36, p_0=0.69, ω_0=0.07, p_s=0.02, ω_S=1.4	157, 159, 171, 198, 314, 318, 324, 353, 364, 368	A1	-3910.5	κ=2.33, p_0=0.64, ω_0=0.06	1.5	0.2207

Table S5. Statistical difference for PEPC Km for PEP, Hill coefficient, and Vmax for species of Suaedoideae without and with glucose 6-P (G6P). Different numbers (1 or 2), without and with G6P, but within the same species indicate a significant difference ($p=0.05$). Different letters (A, B, etc.) among species, but with or without G6P, indicate significant differences ($p = 0.05$). The interaction between species and G6P was highly significant for Km ($P < 0.0001$, $F = 64.01$, df1 = 8, df2 = 5.25), the Hill Coefficient ($P = 0.0014$, $F = 13.60$, df1 = 8, df2 = 6.89), and PEPC Vmax ($P = 0.0008$, $F = 23.98$, df1 = 8, df2 = 5.59). Comparisons were based on the species cell means. Fisher's LSD was used to assess pairwise comparison between the species cell means.

Species	Photosynthetic mode	Amino Acid at residue 733	Amino Acid at residue 780	Km		Hill coefficient		PEPC Vmax	
				No G6P	5 mM G6P	No G6P	5 mM G6P	No G6P	5 mM G6P
S. accuminata	Kranz C_4	M	S	1BC	2AB	1A	2A	1E	2D
S. eltonica	Kranz C_4	M	S	1AB	2A	1ABCD	2B	1D	2C
S. moquinii	Kranz C_4	L	A	1CED	1A	1C	2B	1B	2BC
S. fruticosa	Kranz C_4	L	A	1BCD	2A	1BCD	2B	1CDE	2ABC
S. aralocaspica	Single-Cell C_4	L	A	1BC	2A	1AB	2B	1ABC	2AB
S. linearis	C_3	F	A	1E	2C	1D	1AB	1H	2E
S. physophora	C_3	F	A	1E	2BC	1E	1AB	1F	1E
S. linifolia	C_3	F	A	1D	2BC	1E	1B	1G	1E
Zea mays	C_4	V	S	1A	2A	1D	1A	1A	2A

Table S6. Rubisco large subunit residue polymorphisms among investigated Suaedoideae species. Amino acid replacements putatively associated with increased Rubisco kcatc are highlighted in red.

Species	Section	Photosynthetic mode	Rubisco large subunit residue number																		
			9	32	43	143	145	200	209	225	232	262	266	270	279	281	439	449	461	473	474
Suaeda linearis	*Brezia*	C_3	A	Q	S	A	I	T	Q	L	T	V	M	L	T	A	R	T	I	D	T
Suaeda physophora	*Physophora*	C_3	•	•	•	•	•	•	•	•	•	•	K	•	•	•	•	S	•	•	•
Suaeda linifolia	*Schanginia*	C_3	•	•	•	•	•	•	•	•	•	•	•	•	•	•	•	S	•	•	•
Suaeda vera	*Suaeda*	C_3	•	•	•	•	•	•	•	•	•	•	•	•	•	•	•	S	•	•	•
Suaeda accuminata	*Schoberia*	Kranz C_4	•	•	•	•	•	•	•	•	•	•	•	•	•	•	•	S	•	•	•
Suaeda eltonica	*Schoberia*	Kranz C_4	•	•	•	•	•	S	P	•	•	•	•	•	•	•	•	S	•	•	•
Suaeda altissima	*Salsina*	Kranz C_4	•	•	T	•	V	•	•	•	•	•	•	I	•	•	•	S	•	•	•
Suaeda moquinii	*Salsina*	Kranz C_4	T	•	T	•	V	•	•	•	P	•	•	•	•	S	•	S	•	•	•
Suaeda fruticosa	*Salsina*	Kranz C_4	•	•	T	•	V	•	•	•	•	•	•	•	•	•	•	S	•	•	•
Suaeda aralocaspica	*Borszczowia*	Single-Cell C_4	•	•	•	•	•	•	•	•	•	•	•	•	•	•	•	S	•	•	•
Bienertia cycloptera	*Bienertia*	Single-Cell C_4	•	K	•	S	•	•	•	I	•	A	•	I	S	•	S	A	R	E	P
Bienertia sinuspersici	*Bienertia*	Single-Cell C_4	•	K	•	S	•	•	•	I	•	A	•	I	S	•	S	A	V	E	P

Figure S1. Phylogeny of Suaedoideae taxa used for *ppc-1* positive selection analysis showing key amino acid changes. Suggested role of amino acids are; 364 and 368 glucose-6-phosphate binding, 733 and 780 phospho-enol-pyruvate binding, 868 and 879 malate binding. Clades leading to C_4 photosynthesis are highlighted in red. Phylogeny generated using ITS, *atp-rbcL*, *psbB-psbH*, and *ppc1* third position plus intron sequence. Abbreviations: *B.* = *Bienertia*, *S.* = *Suaeda*.

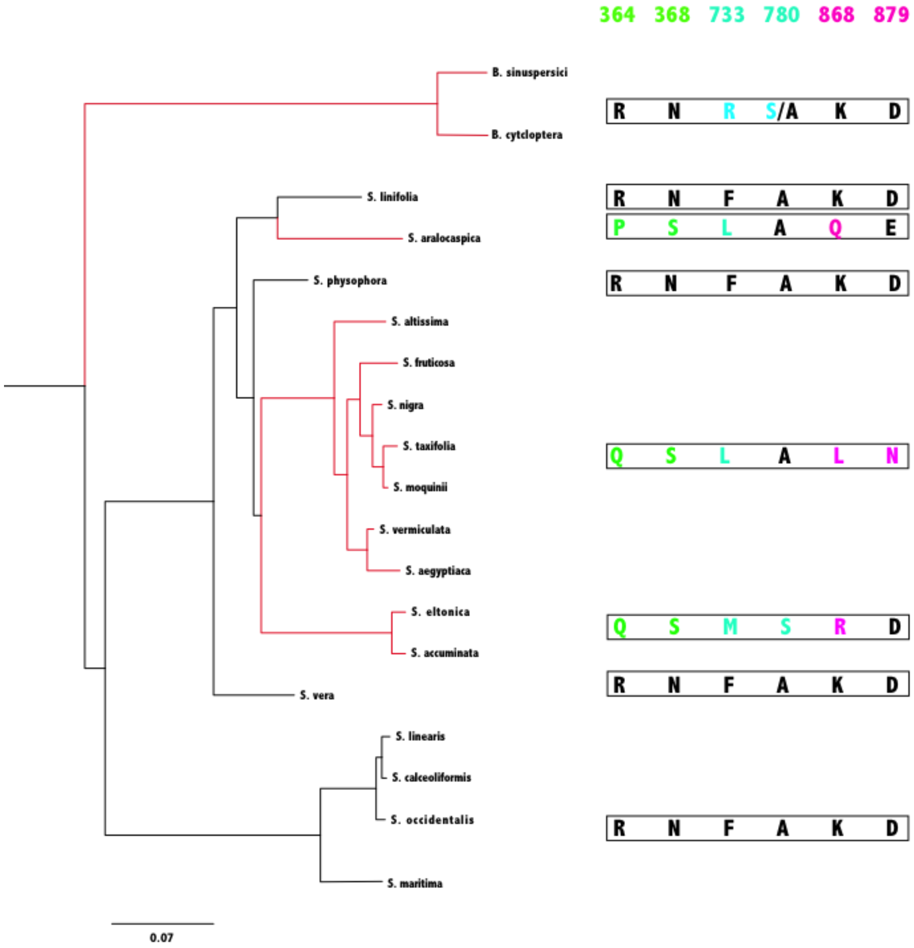

Supplement: Supplementary Data [file supp_erv431_jexbot153007_file001.pdf]
